# A randomised controlled trial to assess the effectiveness of a nurse-led palliative care intervention for HIV positive patients on antiretroviral therapy: recruitment, refusal, randomisation and missing data

**DOI:** 10.1186/1756-0500-7-600

**Published:** 2014-09-03

**Authors:** Keira Lowther, Irene J Higginson, Victoria Simms, Nancy Gikaara, Aabid Ahmed, Zipporah Ali, Gaudencia Afuande, Hellen Kariuki, Lorraine Sherr, Rachel Jenkins, Lucy Selman, Richard Harding

**Affiliations:** Department of Palliative Care and Rehabilitation, Cicely Saunders Institute, King’s College London, Bessemer Road, London, SE5 9PJ UK; London School of Hygiene and Tropical Medicine, London, UK; Kenyan Hospice Palliative Care Association, Nairobi, Kenya; Bomu Hospital, Mombasa, Kenya; Department of Medical Physiology, University of Nairobi, Nairobi, Kenya; Health Psychology Unit, University College London, London, UK; Institute of Psychiatry, Kings College London, London, UK

**Keywords:** HIV/AIDS, Palliative care, RCT, Antiretroviral therapy

## Abstract

**Background:**

Despite the life threatening nature of an HIV diagnosis and the multidimensional problems experienced by this patient population during antiretroviral therapy, the effectiveness of a palliative care approach for HIV positive patients on ART is as yet unknown.

**Findings:**

A randomised controlled trial (RCT) was conducted in a sample of 120 HIV positive patients on ART in an urban clinic in Mombasa, Kenya. The intervention was a minimum of seven sessions of multidimensional, person-centred care, given by HIV nurses trained in the palliative care approach over a period of 5 months. Rates of recruitment and refusal, the effectiveness of the randomisation procedure, trial follow-up and attrition and extent of missing data are reported.

120 patients (60 randomised to control arm, 60 randomised to intervention arm) were recruited over 5.5 months, with a refusal rate of 55.7%. During the study period, three participants died from cancer, three withdrew (two moved away and one withdrew due to time constraints). All of these patients were in the intervention arm: details are reported. There were five additional missing monthly interviews in both the control and intervention study arm, bringing the total of missing data to 26 data points (4.3%).

**Discussion:**

The quality and implications of these data are discussed extensively and openly, including the effect of full and ethical consent procedures, respondent burden, HIV stigma, accurate randomisation, patient safety and the impact of the intervention. Data on recruitment randomisation, attrition and missing data in clinical trials should be routinely reported, in conjunction with the now established practice of publishing study protocols to enhance research integrity, transparency and quality. Transparency is especially important in cross cultural settings, in which the sources of funding and trial design are often not based in the country of data collection. Findings reported can be used to inform future RCTs in this area.

**Trial registration:**

Clinicaltrials.gov NCT01608802.

## Findings

### Background

Since the advent of ART, and more recently since the roll out in sub Saharan Africa (SSA), morbidity and mortality of HIV positive people have been transformed [1]. Healthcare which was mostly focused on symptom control has become focused on virological control, prolonging life and enhancing quality of life for millions of HIV positive people world-wide including within SSA [2]. At the end of 2010, 6.65 million people in low and middle income countries were receiving ART [3]. As international guidelines change, lowering the CD4 threshold for initiation, the proportion of patients and thus the size of the population eligible for ART increases [4, 5]. In Kenya, where this study was conducted, coverage is good and therefore increasing numbers of patients are on ART [6]. This means that there is an increasing population for whom quality of life is now a focus.

Improving access to ART has required a huge adaptation in health care provision: an emphasis on the delivery of ART via rural clinics, the integration and streamlining of services, and investment in minimising loss to follow up of patients who are eligible for ART due to the need for high rates of adherence [3]. While the systemic changes that have come about as a result of the need to scale-up ART provision are laudable, service provision amidst the changing nature of the experience of living with HIV in SSA requires attention.

Although deaths due to AIDS-related illness have decreased due to improvements in treatment and many patients are living longer and in better health, and although two patients have been functionally cured, HIV remains an incurable, life-limiting condition [3]. Increasing numbers of patients are living with HIV with multidimensional problems that include physical symptoms, psychological and spiritual distress as well as social problems such as isolation and stigma [7–11]. A shift in the focus of care is required to address patients’ multidimensional needs while continuing to ensure access to ART, particularly in light of evidence that unaddressed psychological distress and depression are associated with non-adherence to ART and treatment failure [12–14].

Palliative care offers a patient centred, holistic approach to HIV care which aims to improve the quality of life of patients and their families [15]. It integrates physical symptom relief with psychological and spiritual aspects of care, and is mandated throughout the course of life threatening illnesses, in conjunction with other therapies intended to prolong life, such as ART [15, 16]. Previous evidence of the effectiveness of a palliative care approach for HIV has been generated prior to widespread availability of ART; therefore, evidence is needed to identify effective models of holistic care to meet the needs of patients with HIV on ART and their families [17].

We conducted an RCT of a palliative care intervention for patients with HIV infection established on ART. The study protocol has been registered and published (Clinicaltrials.gov no. 6594/3200) [18]. Here we present data regarding recruitment, refusal to participate, randomisation, and missing data. This is particularly important in trial research in cross cultural settings, where there is potentially a greater need for transparency due to concerns around ethical implementation of trials and risks of exploitation when conducting research among vulnerable populations [19].

The trial findings will be reported in a subsequent publication according to guidance from the Consolidated Standards of Reporting Trials (CONSORT) [20].

### Methods

#### Study design

TOPCare, (Treatment Outcomes in Palliative Care) is a mixed method study comprising a randomised controlled trial (RCT) with qualitative interviews among a subset of the trial sample [18].

#### Ethics

Ethics approval was obtained from the relevant bodies, (see section Ethical approval). Participants and their data were handled in strictest confidence. Study files were kept securely in a locked cabinet at the study site, separately from any identifying information.

#### Participants

Participants were consecutively recruited from the HIV clinic at the study site. To begin screening, the researcher, using a random number table, selected a number from within a range corresponding to numbers allocated to register patients currently in attendance at the study site. Initial inclusion criteria were: HIV positive, adult (>18 years old), on ART for more than one month and cognitively able to consent to participation in the study. Participants meeting this criteria were then asked to rate their pain and symptoms on the African Palliative Care Association African POS (APOS), an outcome measure validated in sub Saharan Africa [21, 22]. Participants reporting 3, 4, or 5 on a 0 (no pain)-5 (overwhelming pain) point scale that had endured for at least two weeks were informed about the trial and invited to participate. They were then taken through the full information sheet and consent process in kiSwahili, or English according to participants’ choice.

#### Randomisation

After completion of the baseline data collection interview, block randomisation was performed to allocate participants to control or intervention group. There were three blocks to ensure an even and manageable workload for the study nurses. Slips of paper, (discarded after single use) were blindly selected by the researcher to randomly allocate the participants to a study arm.

#### Standard best practice

The control participants continued to receive standard best HIV care at the comprehensive care clinic. This consisted of nursing or medical assessment, repeat prescription of ART, referrals to ART adherence support, nutrition support or community support team as appropriate. Essential medicines and basic medical care are free to HIV positive patients.

#### The intervention

The nurses delivering the intervention volunteered from the comprehensive care clinic to attend the palliative care training and temporarily become the study nurses. This palliative care training comprised two weeks of didactic training and mentoring in palliative care, provided by trainers from the Kenyan Hospice and Palliative Care Association (KEHPCA). Intervention participants received a minimum of seven appointments with the study nurse over the four- month course of the study. For each appointment the components of standard care were delivered by the intervention nurses in the clinic in addition to the palliative care package. In order to reduce contamination, the intervention nurses did not provide standard care to any participants not in the intervention arm of the study during the trial period.

The palliative nursing assessment was multidimensional, representing the holistic model of palliative care provision, and was informed by similar assessment procedures from sub Saharan Africa [15]. Care was delivered by the study nurses in accordance with the African Palliative Care (APCA) Standards for providing quality palliative care [23]. Support and supervision was provided by the local hospice, which managed complex participants with needs beyond the competency of the study nurses. Participants were also referred to members of the hospital multidisciplinary team, such as chaplaincy or nutritional support if appropriate.

#### Data collection interviews

Interviews were conducted face to face with questions read aloud and questionnaires completed by a trained local researcher, to account for low literacy rates among this population. Interviews were conducted in kiSwahili or English at monthly intervals for 4 months post baseline (Table 1).

**Table 1:** **Chronology of data collection and delivery of intervention versus standard care**

	Study event
Baseline	● ◊ ∆
Within 1 week of first contact	◊
Within 2 weeks of first contact	◊
Month 1	● ◊ ∆
Month 2	● ◊ ∆
Month 3	● ◊ ∆
Month 4	● ◊ ∆

● = Data collection interview.

◊ = Study nurse session for intervention participants (minimum).

∆ = Clinic nurse session for control participants (minimum).

#### Outcome measures

We used self report questionnaires to assess patient outcomes: the A-POS) [21], the Medical Outcomes Study-HIV (MOS-HIV) [24], the General Health Questionnaire-12 (GHQ-12) [25, 26], Client services receipt inventory (CSRI) [27] and questions about adherence and risk behaviours. Decisions about which questionnaires to use were based on the availability of validation data in similar populations.

#### Analysis

Objectives of the analysis were to: 1) examine recruitment flow 2) describe demographic characteristics of the two trial arms, 3) determine refusal rate, 4) examine the distribution of patient attrition and missing data. A CONSORT diagram is presented detailing recruitment flow, refusal rate and attrition at each monthly time point [20]. Baseline data were examined for differences in distribution of variables between intervention and control groups.

Missing data analysis examined the pattern of missing data and compared the clinical and demographic characteristics of participants who withdrew prematurely from the study with the remainder of the sample with complete data.

#### Sample size

A sample of 120 participants was required to meet the RCT objectives [18]. Based on the opinion of the study site clinic manager regarding numbers of participants attending the clinic, it was estimated that the researcher could recruit one patient meeting the inclusion criteria per day, and therefore recruitment would last approximately 6 months.

### Results

#### Recruitment flow

A total of 2070 participants were screened over the recruitment period, which lasted 5.5 months. Of all participants who were eligible for the inclusion in the trial, 55.72% refused to consent to participation (n = 151). Of the screened clinic population who were adult, HIV positive, 1664/2070 (80.4%) were on ART for more than one month, 271/1664 (16.3%) reported moderate to overwhelming pain or symptoms lasting more than two weeks and 120/271 (44.3%) consented. Data on reasons for refusal was not formally collected, but informally the researcher was informed that participants often had difficulty taking time off work to attend the data collection interviews.

#### Randomisation

The remaining 120 consenting participants were randomised to receive either the intervention (n = 60) or the control (n = 60). Sample characteristics are compared in Table 2.

**Table 2:** **Sample characteristics for whole sample and by control and intervention study arm at baseline**

	Entire sample (n = 120)	Control (n = 60)	Intervention (n = 60)
Female n (%)	97 (81)	49 (82)	48 (80)
Mean age in years (sd, range)	39 (8.9, 22–64)	40.5 (9.2, 22–64)	38.3 (8.2, 23–60)
Has a partner (% yes)	63	60	66.7
Median no. children (IQR)	2 (1–4)	2.5 (1–4)	2 (1–4)
Median no. financial dependents (IQR)	3 (2–5)	4 (3–5.5)	3 (2–5)
Education			
Never been to school	10	6	4
<4 yrs of school	3	1	2
Primary	76	35	41
Secondary	27	17	10
Diploma	4	1	3
**Poverty quintile (5 quantiles of wealth), n (%)**			
1	26 (22.2)	14 (23.3)	12 (21.1)
2	21 (18.0)	9 (15.0)	12 (21.1)
3	23 (19.7)	9 (15.0)	14 (24.6)
4	24 (20.5)	12 (20.0)	12 (21.1)
5	23 (19.7)	16 (26.7)	7 (12.3)
Median years since HIV diagnosis (IQR)	3.5 (1.3–5.2)	4.7 (2.4–5.7)	2.6 (0.9–4.4)
Median years on ART (IQR)	2.5 (0.8–4.2)	3.0 (1.6–5.0)	1.6 (0.4–3.5)
Median CD4 count (cells/mm^3^, IQR)	358 (223–506)	343 (209–558)	359 (247–490)
Receiving TB treatment (% yes)	8	12	5
WHO stage			
Stage 1	13	4	9
Stage 2	41	22	19
Stage 3	62	32	30
Stage 4	4	2	2

On average control arm participants had been diagnosed and on ART for longer than intervention arm participants.

Baseline outcome data (mental and physical health summary score from MOS-HIV, GHQ-12 score and A-POS total score), not reported in Table 2 also indicated an even distribution across intervention and control study arms.

#### Trial follow up and attrition

**Figure 1:**
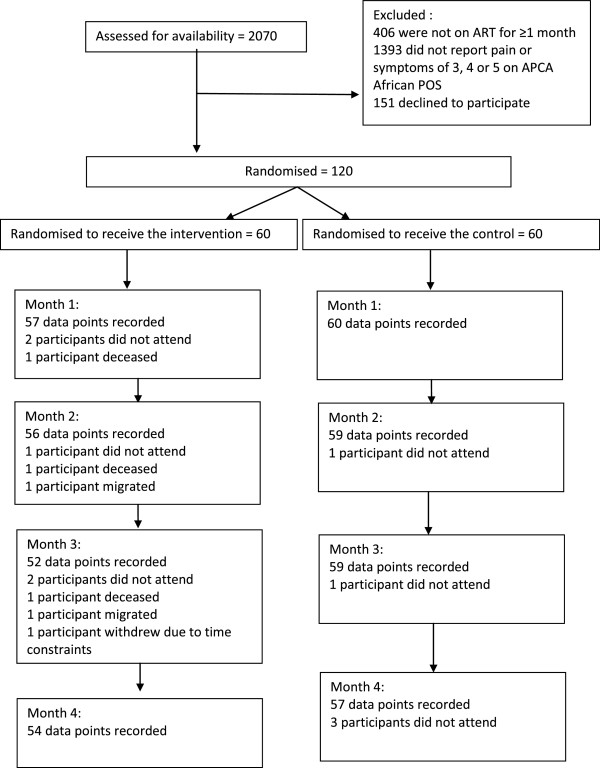
**CONSORT diagram showing flow of participants through the study.**

The recruitment and follow up flow of the study participants is depicted in a CONSORT diagram in Figure 1 [20]. Over the trial period there were 6 participants who prematurely withdrew from the trial, (all allocated to the intervention arm) representing 5% of the total sample (n = 120). Three participants died before the trial was complete, three withdrew prematurely (two moved away and one became less available due to work commitment). Of those participants who remained alive during the study period, 98.1% of possible data was obtained.

#### Missing data

Within the interviews which were attended, there was only one missing item (omitted or skipped question within the completed questionnaires). All other attended interviews had complete data. A total of 26 interviews were not attended, therefore for these interviews there is no data for any outcome measure (26 missing interviews out of a possible total of 600: 4.3% missing). The distribution and time line of this missing data is described in Table 3.

**Table 3:** **Summary of missing interviews by time point and study arm**

	Month 1	Month 2	Month 3	Month 4
Total control missing data	0	1	1	3
Total intervention missing data	3	4	8	6
Cumulative percentage of missing data	2.5%	3.3%	4.7%	4.3%

Whilst the figure of 4.3% missing data is low, of this 4.3%, 21 of the 26 missing interviews (81%) occur in the intervention arm. The distribution of missed single interviews is evenly distributed across control and intervention study arms (five missing single interviews in each). The remaining missing data is from participants who missed more than one interview and prematurely withdrew from the study, who were all in the intervention arm.

The clinical and demographic details of participants who exited the trial before the final time point are examined in Table 4. The table contains the data for three participants who died (attrition due to death or ADD) and two who left the area (attrition at random AaR) [28]. The remaining participant left the study due to unavailability.

**Table 4:** **Demographic and clinical characteristics of participants who exited the trail before the final time point**

Missing patients number	1	2	3	4	5	6	Whole sample (n = 120)
Reason for exit	Deceased	Deceased	Deceased	Withdrew	Withdrew	Withdrew	
Number of data points	1	3	2	3	2	3	5
Gender	f	f	f	m	f	m	80% female
Time since diagnosis (years)	5	<1	<1	<1	<1	<1	Median 3.5 (IQR 1.3–5.2)
Time on ART (years)	5	<1	<1	<1	<1	<1	Median 2.5 (IQR 1.8–4.2)
Poverty quintile (1 = higher, 5 = lower wealth)	5	3	1	1	4	4	n/a
Receipt of TB treatment	no	no	no	no	no	no	8% yes
CD4 at baseline	358	524	403	473	492	322	Median 358 (IQR 223–506)
Mental health summary score (MHSS) at baseline (MOS-HIV) (1–100 range)	12.95	36.79	56.80	20.72	48.85	44.78	Median 44.8 (IQR 36.97–53.78)
Physical health summary score (PHSS) at baseline (MOS-HIV) (1–100 range)	15.38	15.94	47.20	26.49	59.60	58.92	Median 45.57 (IQR 33.51–53.15)
GHQ-12 score at baseline (Chronic scoring method, 0–12; 12 worst)	11	12	0	8	3	8	Median 6 IQR (3–9)

The sub sample of participants who exited before the final time-point (n = 6) had been diagnosed more recently and had received ART for a shorter time compared with the sample median, but otherwise there appeared to be no strong evidence of difference between these participants and those who remained in the study. Due to the small numbers, it was not possible to perform analysis to identify whether there was any statistically significant difference between those lost to follow up and the remainder of the sample.

The minimal amount of missing data is largely due to the efforts of the researcher, who worked in close collaboration with the staff and management at the study site to contact participants who had not attended their data collection appointments. Community tracers from the comprehensive care clinic, usually employed to ensure adherence to ART, were asked to enquire about participants who had not attended their appointments using their contacts and local knowledge. This collaborative approach contributed to the minimal amount of missing data and subsequent high quality dataset.

It is important to closely examine the participant data to identify whether the participants who exited the trial before the final time point, left or died due to their participation in the trial, particularly as those who left prematurely were allocated to the intervention study arm. We have performed such an examination and are satisfied that the attrition and missing data is not due to receipt of the intervention, as there were extraneous circumstances in each case. While a detailed report of each relevant participant would compromise patient anonymity, the salient points can be summarised as follows:

Two participants who died in the intervention group were diagnosed with advanced cervical cancer, one during the trial, and one who had previously been diagnosed. Management of their cancer was in line with hospital policy; if eligible for chemotherapy and unable to afford it, they would both have been able to apply for a hardship fund held by the hospital, ensuring access to treatment. These participants received education and counselling on topics such as nutrition, HIV, ART adherence, hygiene and medication side effects in addition to psychosocial support for themselves and their families. The additional support they received due to their participation and allocation to the intervention arm consisted of pain control medication including opioids, family support, and information during the dying process.

Three participants migrated before completing the study. One subsequently died of malaria in the rural area she had moved to before the study ended. The final patient who withdrew from the study prematurely became unavailable due to work commitments.

### Discussion

This RCT of a nurse-led palliative care intervention is the first of its kind for HIV positive participants on ART in the field of palliative care research, and represents an ambitious attempt to provide evidence of effectiveness for a holistic approach to the delivery of HIV/ART care in sub Saharan Africa. In the current climate of ART roll out in the region, and massive increase in coverage of access to ART, it is important not to neglect the holistic needs of the patient, both for patient quality of life and for efficient and effective adherence to ART.

#### Screening

During recruitment, the inclusion criteria revealed 16.3% of HIV positive patients on ART attending the clinic had moderate to severe pain or symptoms. This reflects the relative physical health of these patients who have been on ART for more than one month. The relatively strict inclusion criteria were to identify those in pain or with symptoms at recruitment, as change in reported pain score was the primary outcome of the trial.

Anecdotally the study nurses, on return to their usual role in the HIV clinic reported that their skills in palliative care had enabled them to detect high levels of psychosocial distress in many patients. They expressed surprise that they identified patients in the clinic who reported being physically well, attending only for refill of their ART prescription, who were found to have severe unresolved emotional pain.

#### Consent and refusal

The refusal rate (55.72%) appears to be relatively high compared with studies in similar populations in Sub Saharan Africa (30% [29], 12% [30], 3% [31]). A study conducted in the same region of Kenya as the current trial explored the concept of informed consent in a trial with 100% consent rate [32]. The study authors identify that many participants consent to participation due to fears of inferior treatment if they refuse. Other reasons for consenting to participation included participants who assumed that there would be direct therapeutic benefit, despite the randomised controlled trial study design [32]. This paper also reported that if participants refused to participate, often it was because they anticipated little personal therapeutic benefit for the personal cost to them, either financially or in time [32]. In the light of these findings, our comparatively high refusal rate of 55.7% may not be a cause for concern.

The researcher in the TOPCare study was instructed to guide the participants through the information sheet slowly, answer questions and to specifically advise participants that there would be no consequences for refusal to participate. She was also instructed to explain clearly that if the participant was randomised to the control arm, they would continue to receive the same care in the clinic as previously but would be expected to attend five data collection interviews, each lasting approximately 45 minutes. This could have contributed to the high refusal rate by ensuring that the participants had a good understanding of the costs and benefits of participation, financial and otherwise, and the very real possibility that they may not receive the intervention.

Research into the process of informed consent in Ethiopia found that having a stigmatising condition reduced the likelihood of consent, as participants feared further stigmatisation as a result of their participation [33]. Stigmatisation is a major concern for participants with HIV in Kenya, as documented by the extensive literature on the subject [34–37]. It is therefore likely to have contributed to the high refusal rate.

Respondent burden is defined as a subjective phenomenon of physical, psychological or financial hardship associated with participation in research [38]. Examples are when questions may be sensitive, stressful to answer or the process could be demanding in terms of interview time or frequency. Research fatigue is elsewhere identified as associated with a lack of practical change seen resulting from previous research engagements [39]. It is possible that the participants felt that this research was too costly, either in terms of time taken, financially due to missed work opportunities or emotionally due to the sensitive nature of the subject. It is important to bear in mind that this study was conducted in an outpatient setting, where participants have roles and responsibilities to fulfil outside of the study setting. The study site for this trial is very experienced in conducting research and it is a possibility that this population are very research experienced, increasing the chance of respondent burden or fatigue.

Therefore, high refusal rate in this study could be due to a combination of the clear explanation of the cost in terms of patient time and potential lack of benefit if allocated to the control study arm, the stigmatising nature of HIV and research fatigue. Unfortunately no data were formally collected on reasons for refusal to participate.

While 55.7% of all eligible participants in the clinic approached for consent refused to participate, this refusal rate should be examined in the context of the minimal attrition and missing data in the dataset from this trial. It is possible that due to the clear explanation to participants at consent, participants were aware of the costs to them and therefore did not consent if they would be unable to attend and participate fully in the study. In addition, the researcher paid close attention to participants who did not attend for interview and went to great lengths to contact them and encourage them to participate. Because of the value placed by the study design team on a clear ethical approach, it was ensured that participants had access to culturally appropriate consent materials and a kiSwahilli speaker to discuss the costs and benefits to the patient in detail. These elements of the procedure may have inflated the refusal rate but achieved an ethical recruitment process with low attrition.

#### Missing data distribution

There were three patients within the sample who died during the study period. All had been allocated to the intervention arm. There are five possible reasons for this.

The first is that the randomisation procedure was tampered with. In response to these findings, we reviewed the randomisation procedure with the researcher, and examined the research and clinical care data for each patient who withdrew prematurely. Prior to data collection, the researcher was trained in the importance of the randomisation procedure in gaining good evidence, and taught that without which we could not identify and then advocate for more effective treatment for this patient group. The researcher was then closely supported, with weekly monitoring of data throughout recruitment and randomisation, in addition to a short period of direct observation. During training, the research team discussed the difficulties they would face, such as randomisation of sick participants to the control arm, and through role play researchers were prepared for this eventuality to prevent compromising the randomisation process. Thus, we believe that the randomisation procedure was performed accurately, with no deviation from protocol.

Another possibility is that the intervention shortened the lives of participants. Availability of chemotherapy for cancer care in East Africa is recognised to be inadequate, primarily due to financial constraints [40]. Whilst there is a hardship fund available in the hospital for those who are unable to afford chemotherapy or radiotherapy, this would not have benefitted our patients with advanced cancer, regardless of their inclusion in a study. Two were too advanced in their disease for these therapies to be of benefit and the third would have not been eligible, because of his stable financial status. It is of note that since this research, the hospital has begun a screening programme for cervical and breast cancer, due to the high numbers of patients presenting with advanced disease.

Theoretically it is also possible that intervention patients were denied some aspect of the care which was available to control patients; e.g. the pharmacy was closed by the time they had finished their palliative care appointment. In reality this did not happen as the palliative care nurses were extremely diligent at ensuring that their patients received all that they needed in terms of nursing care and medical input. They carefully followed up on results of investigations, and followed up referrals to physicians, chaplains, imams and hospice.

A third potential reason for a death was that the patients who disclosed that their husbands were abusive could have been put in jeopardy, if their husbands discovered that others in authority were aware of the abuse. However, the nurses were extremely proficient and as a professional standard, would not disclose this to anyone outside of the small palliative care team. The team were in the process of facilitating couples counselling for both patients who were having difficulties in their marital relationship. Their role was supportive, building the communication skills and confidence of the women in this situation so they could advocate for themselves and seek further support if they felt it necessary. As the community were able to confirm that the patient with the abusive husband who reportedly died, had travelled to her family farm, it is not thought that she came to any direct harm due to a disclosure of abuse. The other patient volunteered the information to the study team that she was migrating to the rural area due to her husband leaving her.

A fourth possible concern was that, as these patients with severe pain were receiving opioid based analgesia, which is uncommon in Kenya, they could have received an overdose, leading to their death. The palliative care nursing team received intensive support and training in identifying patients who were in need of opioid analgesia. The hospital did not provide this medication, so it was provided by the local hospice and supported by their staff, who carefully monitored each patient closely. There were only three patients who received opioid analgesia during the study period, enabling this to be closely scrutinised and controlled.

The final option is that all the deaths occurred in the intervention group by chance.

In our forthcoming report of the study findings, analysis will be conducted including these participants with partial data as it is anticipated that due to the low numbers, the impact on findings will be minimal.

#### Randomisation

Because of unreliable internet connectivity at the study setting, electronic randomisation was not possible. The randomisation approach that we used appears to have been effective, with even distribution between study arms of almost all variables.

It is possible that in the future, recruitment in this population will remain challenging, as participants see themselves as healthy because of their ability to be economically productive, normalising their pain and symptoms. However, these methodological difficulties should not preclude high quality research, as evidence on the prevalence of pain, symptoms and psychological distress in this population, indicate that multidimensional problems in the participants group continue in the presence of ART, and should be addressed [7, 14, 41].

### Conclusion and recommendations

In publishing this data, we restate our commitment to ethics and quality in research, in addition to preparing the data for analysis of outcomes. Analysis shows full recruitment of the planned sample was successful, with good retention and minimal attrition. We have established that recruitment procedures were appropriately implemented, missing data is low, and there are few baseline differences between arms.

In addition, our relatively high refusal rate may indicate that informed consent has taken place, particularly when accompanied with low participant attrition. This reflects our commitment to a clear and ethical recruitment procedure.

We have found that it is feasible to successfully and ethically conduct an RCT, in a resource-constrained setting with a strong collaborative relationship. We propose that researchers conducting research in cross cultural contexts continue to report their experiences in this way, including difficulties and unexpected findings, to increase research transparency, integrity and quality and to allow for better interpretation of the forthcoming outcome data.

### Ethical approval

Ethical approval was obtained from King’s College London Research Ethics Committee (BDM/10/11-31) and the Kenyan Medical Research Institute (KEMRI/RES/7/3/1).

## Abbreviations

AaR: Attrition at random
ADD: Attrition due to death
APCA: African palliative care association
APOS: APCA palliative outcomes scale
ART: Anti retroviral therapy
CONSORT: Consolidated standards of reporting trials
CSRI: Client services receipt inventory
GHQ-12: General household questionnaire
HIV: Human immuno-deficiency virus
KEHPCA: Kenyan hospice and palliative care association
MOS-HIV: Medical outcomes study-hiv
RCT: Randomised controlled trial
SSA: Sub saharan Africa
TOPCare: Treatment outcomes in palliative care.
